# Unraveling the Mitogenomic Characteristics and Phylogenetic Implications of *Leuciscus merzbacheri* (Zugmayer, 1912), an Endangered Fish in the Junggar Basin of Xinjiang, Northwest China

**DOI:** 10.3390/genes15101284

**Published:** 2024-09-30

**Authors:** Yan Sun, Tianyan Yang

**Affiliations:** Fishery College, Zhejiang Ocean University, Zhoushan 316022, China; sy1249032764@163.com

**Keywords:** Illumina sequencing, mitogenome, phylogenic relationship, Leuciscinae

## Abstract

Background: *Leuciscus merzbacheri* is a rare and endangered fish in Xinjiang, China. As a representative species of the fauna in the Junggar Basin, it is of high economic and scientific value. The genetic data are still limited, and the mitochondrial genomic characteristics remain unexplored. Methods: A high-throughput sequencing method was used to obtain the complete mitogenome of *L. merzbacheri*. Results: The full length of the circular DNA was 16,609 bp, and it consisted of 13 protein-coding genes (PCGs), 22 tRNAs, 2 rRNAs and 2 non-coding regions. The overall nucleotide compositions of both the mitogenome and PCGs showed an obvious AT preference with percentages of 54.20% and 53.60%, respectively. Three commonly used amino acids were Leu (16.43%), Ala (8.95%) and Thr (7.85%) in turn. All tRNAs could form the typical clover structures excluding tRNA-Ser ^AGY^. The presumed secondary structures of two rRNAs contained several stem-loop domains, and the structure of 12S rRNA seemed to be more stable than that of 16S rRNA. Extended termination sequence regions (ETASs), central conserved regions (CSB-F, CSB-E and CSB-D), and conserved sequence regions (CSB-1, CSB-2 and CSB-3) were identified in the control region. The phylogenetic tree showed that *L. merzbacheri* was recovered with strong supports as a sister to the other members of the genus. The location in the outermost branch implied that it might be a relatively ancient species among its congeners. Conclusions: This study would complement the genetic data on *L. merzbacheri* and contribute to a better understanding of molecular evolution in *Leuciscus* as well.

## 1. Introduction

*Leuciscus* fishes are widely distributed in rivers and lakes in cold-temperate plain areas of Eurasia [1]. The number of species or subspecies within the genus *Leuciscus* distributed in China nearly accounts for 25% of the world’s daces [2]. *Leuciscus merzbacheri* is an indigenous fish unique to the Xinjiang Uygur Autonomous Region and only inhabits in the Junggar Basin in the north of Tianshan Mountains, which makes it a landmark species of the fish fauna in this basin [3]. It formerly was the major economical fish of the local areas [3]. However, because of the ecological environment changes and the strengthening of human activities, its habitat is seriously destroyed and the population resource is shrinking continuously [3,4]. Thus, early in 1998, this native fish was listed as a vulnerable species in the “China Red Data Book of Endangered Animals (Pisces)” [5]. Nowadays, it has been considered first-class protected aquatic wildlife in Xinjiang and is also included in “The IUCN Red List of Threatened Species” as a vulnerable species [6]. So far, studies on *L. merzbacheri* have mostly concentrated on the fields of morphological and biological characteristics [7,8,9,10], resource distribution [3,11] and artificial reproduction and protection [12,13]. A finite number of genetic references is available for this rare and endangered fish species, and only the chromosome karyotype and tissue-specific expression of isozymes have been reported until now [14,15]. At the molecular level, only a draft mitogenome and some mitochondrial and nuclear gene fragments, including mitochondrial cytochrome b (Cyt *b*), the control region and the β-actin gene promoter, were sequenced and analyzed [16,17,18,19], which seriously prevented the genetic conservation and utilization of this critically valuable species.

The mitochondrion is an important place for cell respiration, and the adenosine triphosphate (ATP) produced by it can supply energy for life activities [20]. This semiautonomous organelle possesses the only genetic material outside the nucleus and has the capabilities of independent replication, transcription and translation [21,22]. Mitochondrial DNA (mtDNA) is the physical embodiment of the genetic information encoded in the mitochondrion, and this covalently closed double-stranded circular molecule owns specificities, such as maternal inheritance, a lack of recombination, a high copy number and a rapid evolutionary rate [23]. It has become an effective tool for population genetics, molecular phylogenetics, species identification and forensic science [24,25,26]. However, the limited mitochondrial DNA data confine the genetic and taxonomic studies on *L. merzbacheri*. Only Hai et al. estimated the divergence time of Leuciscinae in Xinjiang based on mtDNA Cyt *b* sequences, which revealed that *Leuciscus* originated at the Miocene, and the taxonomic relationship of *L. merzbacheri* was relatively distant from *L. baicalensis*, *L. idus* and *L. waleckii* [27]. Furthermore, its taxonomic status within the genus *Leuciscus* is also confused because of higher species diversity and morphological similarity and even introgressive hybridization in natural homoploid fishes [28,29,30].

In this study, the complete mtDNA genome of *L. merzbacheri* was sequenced, assembled and annotated. Meanwhile, the phylogenetic tree was constructed using the complete mitogenome sequences of the Leuciscinae species published in the GenBank database to provide reference for its classification status. The results will help to promote future studies on the conservation genetics and evolutionary biology of this native and threatened species and other fishes in *Leuciscus*.

## 2. Materials and Methods

### 2.1. Sampling, DNA Extraction and High-Throughput Sequencing

**Figure 1:**
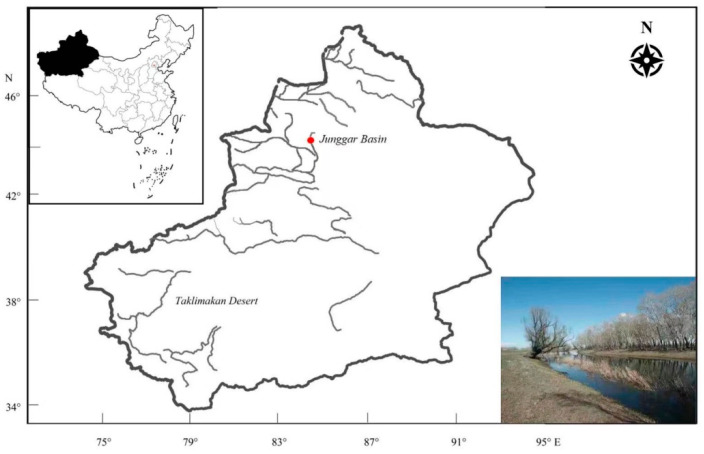
Sampling site of *L. merzbacheri* in this study.

The sample of *L. merzbacheri* was collected in October 2021 from a tributary of the Manas River, which originates from the northern slope of the Tianshan Mountains in Xinjiang, China (Figure 1). A small amount of pectoral fin tissue was clipped and stored in absolute ethanol immediately. The genomic DNA was extracted using the standard phenol–chloroform method [31]. The integrity and purity of DNA were detected via 1% agarose gel electrophoresis and with a Qubit fluorometer, respectively. The qualified template DNA was sheared into fragments of a specific size (300–500 bp) via ultrasonic mechanical interruption (Covaris M220, Woburn, MA, USA), and then, the fragmented DNA was processed through purification, terminal repair, A-tail and sequencing adaptor addition and fragment size selection using agarose gel electrophoresis. Subsequently, the appropriate DNA fragments were amplified via PCR to construct the paired-end library. The prepared library was sequenced on an Illumina HiSeq based on sequencing by synthesis (SBS) technology.

### 2.2. Mitochondrial Genome Assembly and Annotation

Prior to assembly, the raw data were purified by removing adaptors and primers, as well as low quality reads with a quality score lower than 20 (Q < 20) by using Cutadapt 4.4 software [32]. MITObim 1.9.1 software was applied to assemble the mitochondrial genome with default parameter settings based on the clean reads mapped to the reference mitogenome of *L. baicalensis* (GenBank accession No. KF673863) [33]. The obtained mtDNA sequence was annotated using the web-based tool MitoAnnotator (http://mitofish.aori.u-tokyo.ac.jp/annotation/input.html; accessed on 20 February 2024). The mitogenome map of *L. merzbacheri* was plotted using OGDRAW software 1.3.1 [34]. The entire mitogenome sequence was deposited into the Nucleotide database of the National Center for Biotechnology Information (NCBI) under the accession number OR992052. 

### 2.3. Mitogenome Sequence Analysis

The online tools tRNAscan-SE (http://lowelab.ucsc.edu/tRNAscan-SE/; accessed on 5 March 2024) and tRNAdb (http://rna.tbi.univie.ac.at/forna/; accessed on 6 March 2024) were utilized to locate and visualize the cloverleaf structures of tRNA using the default search modes, respectively. The secondary structures of two rRNAs were prognosticated using the R2DT tool of the RNAcentral website (https://rnacentral.org/ (accessed on 10 March 2024)). The location of origin of the L-strand replication region (O_L_) was identified via sequence homology alignment, and its secondary structure was predicted online with Mfold (http://www.unafold.org/; accessed on 15 March 2024). Nucleotide sequences were aligned with MUSCLE 3.8 [35]. MEGA 6.0 software was used to analyze the nucleotide content, amino acid composition and relative synonymous codon usage (RSCU) of the protein-coding gene (PCG) sequences [36]. The formulas AT-skew = (A − T)/(A + T) and GC-skew = (G − C)/(G + C) were used to describe the overall patterns of nucleotide composition in mtDNA sequences [37].

### 2.4. Phylogenetic Analysis

A total of thirty-three complete mtDNA sequences of Leuciscinae species were downloaded from the GenBank database for phylogenetic inference (Table S1). In addition, largemouth bass *Micropterus salmoides* (GenBank accession No. OL339398) belonging to the order Perciformes and family Cehtrachidae was adopted as the outgroup taxa of the evolutionary tree. The trimmed PCG sequences (without stop codons) were concatenated into a matrix with FASconCAT-G 1.04 software [38]. The same optimal nucleotide substitute model “GTR + I + G” for the maximum likelihood (ML) and Bayesian inference (BI) analyses was selected by Modeltest 3.7 software under the Akaike information criterion (AIC) and Bayesian information criterion (BIC), respectively [39]. The ML phylogenetic tree was construct using PhyML 3.0 software, and the tree topology was evaluated with 1000 bootstrap replicates [40]. The BI phylogenetic tree was constructed using Mrbayes 3.2.6 software, with one cold and three heated Markov Chain Monte Carlo (MCMC) chains running for 1,000,000 generations sampled every 1000 generations, and the first 25% of the samples were discarded as burn in [41].

## 3. Results

### 3.1. Mitogenome Structure and Nucleotide Composition

The complete mitogenome of *L. merzbacheri* was 16,609 bp in length, including 13 PCGs, 22 transfer RNAs (tRNAs), 2 ribosomal RNAs (12S rRNA and 16S rRNA) and 2 non-coding regions (D-loop and O_L_) (Table 1, Supplementary Figure S1). Among the 37 genes, only 9 genes (tRNA-Pro, tRNA-Glu, *ND6*, tRNA-Ser ^UCN^, tRNA-Tyr, tRNA-Cys, tRNA-Asn, tRNA-Ala and tRNA-Gln) were located on the light strand (L-strand), and the remainders were encoded by the heavy strand (H-strand). In particular, all PCGs except for *ND6* were situated on H-strand. A total of 7 gene overlaps (2–7 bp) and 11 gene spacers (1–13 bp) were detected in the whole mitochondrial genome. The largest overlaps were between *ATP8* and *ATP6*, as well as *ND4L* and *ND4*. Meanwhile, the longest intergenic gaps appeared between tRNA-Asp and *COII* with the length of 13 bp.

As a remarkable feature of animal mitochondrial genomes, the asymmetry in nucleotide composition of the whole mtDNA was discovered as follows: A 27.90%, G 18.68%, T 26.30% and C 27.12%, showing an obvious A+T bias (54.20%). The patterns of nucleotide distribution throughout the whole mitogenome are exhibited in Supplementary Table S2. The results showed a positive AT-skew value (0.0295) and a negative GC-skew value (−0.1843), respectively. The GC-skew values of 13 PCGs and 2 rRNAs were all less than zero, which illustrated that the nucleotide compositions were heavily anti-bias toward guanine. A conspicuous anti-T bias was observed in *ND6*, which was quite unlike other PCGs and might be related to different coding strands. The D-loop region had the most obvious anti-GC skew of all, accounting for 62.76% of A+T content, and the AT proportion in *ATP8* was also very high, reaching 61.21%. However, only the A+T contents of *ND2* and *ND6* were lower than those of G+C contents, showing a greater abundance of cytosine.

### 3.2. Protein-Coding Genes, Amino Acids and Codon Usage Pattern

The mitochondrial genome of *L. merzbacheri* contained 13 PCGs with a total length of 11,421 bp, which occupied 68.76% of the entire mitogenome. Twelve PCGs were located on the H-strand, but the *ND6* gene was the only exception, which was encoded on the opposite strand. There were two types of initiator codons used by PCGs, in which ATG most frequently appeared, and only *COI* started with GTG. Five genes (*COI*, *ND1*, *ND4L*, *ND6* and *ATP8*) employed the conventional stop codons TAA and TAG. *ATP6* and *ND4* used TA- as the terminal codon, while five genes (*ND2*, *COII*, *COIII*, *ND3* and *Cytb*) used T-- as the terminal codon. 

MEGA6.0 software was used to obtain the RSCU values of 13 PCGs and analyze the codon usage preferences (Table 2). The codon CGA(R) had the highest frequency of RSCU (1.89) with the count number of 87, followed by the codon CCC(P), while the lowest RSCU value 0.32 was found in UCG(S). In general, the numbers of NNT (16) and NNC (16) codons were higher than NNA (14) and NNG (14) codons in the mtDNA PCGs of *L. merzbacheri*, which was consistent with the nucleotide compositions of all 13 PCGs. The amino acid composition was analyzed, and the higher coding frequencies of PCGs were Leu (16.43%), Ala (8.95%) and Thr (7.85%), respectively (Supplementary Figure S2). Among the six available codons for the amino acid leucine, the CUA codon had the highest RSCU value (1.84) within the coding regions analyzed in *L. merzbacheri*. 

### 3.3. Characterization and Structural Prediction of tRNAs and rRNAs

**Figure 2:**
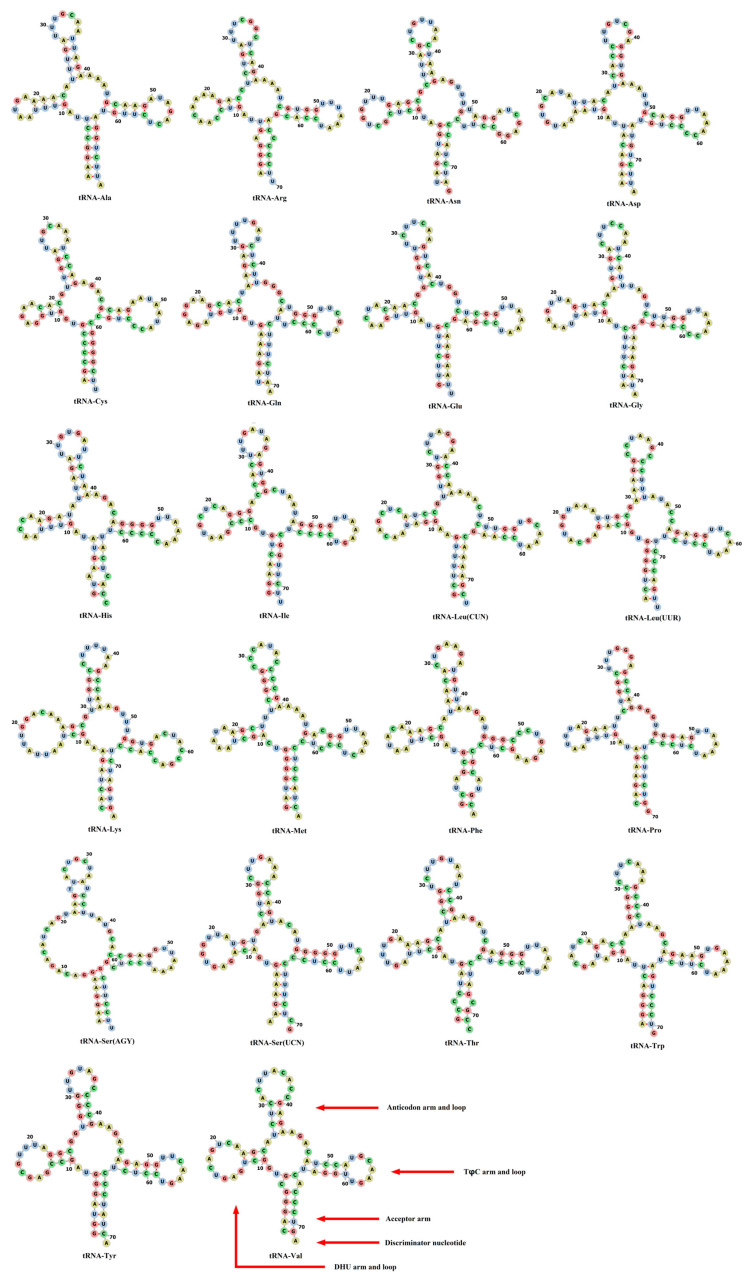
Secondary structures of mitochondrial tRNAs in *L. merzbacheri*.

Twenty-two tRNAs were interspersed among the mitochondrial genes of *L. merzbacheri*, with the length ranging from 67 to 76 bp. Most tRNAs were encoded by the H-strand, except for tRNA-Cys, tRNA-Ala, tRNA-Glu, tRNA-Pro, tRNA-Gln, tRNA-Tyr, tRNA-Ser ^UCN^ and tRNA-Asn. There were a total of 8 gene overlaps or spacers among the 22 tRNA genes. The largest intergenic spacer and overlap were between tRNA-Ser ^UCN^ and tRNA-Asp, as well as between tRNA-Ile and tRNA-Gln, respectively. When predicting the secondary structures of tRNAs, it was found that only tRNA-Ser ^AGN^ did not form the typical clover structure due to the absence of a dihydrouracil (DHU) arm, while the remaining tRNA genes were all clover shaped (Figure 2). In addition, a total of 46 noncanonical base pairs were detected in 20 tRNAs excluding tRNA-Leu ^CUN^ and tRNA-Lys, of which the most abundant type of single-base mismatch was G-U.

The two rRNA genes, 12S rRNA (959 bp) and 16S rRNA (1691 bp), were typically separated by tRNA-Val, and both of them were encoded by the H-strand. The A+T content of 16S rRNA (54.64%) was slightly greater than that of 12S rRNA (50.99%). The secondary structure prediction and visualization of two rRNAs are displayed in Supplementary Figure S3. It was found that 12S rRNA consisted four domains, of which domain I and domain II were variable regions, whereas domain Ⅲ and domain IV were conserved regions [42]. However, the secondary structure of 16S rRNA contained six domains, and each domain was distinguished by a single-strand sequence. The first four domains (I–IV) were variable regions, while the last two domains (V–VI) were conserved regions.

### 3.4. Non-Coding Regions

**Figure 3:**
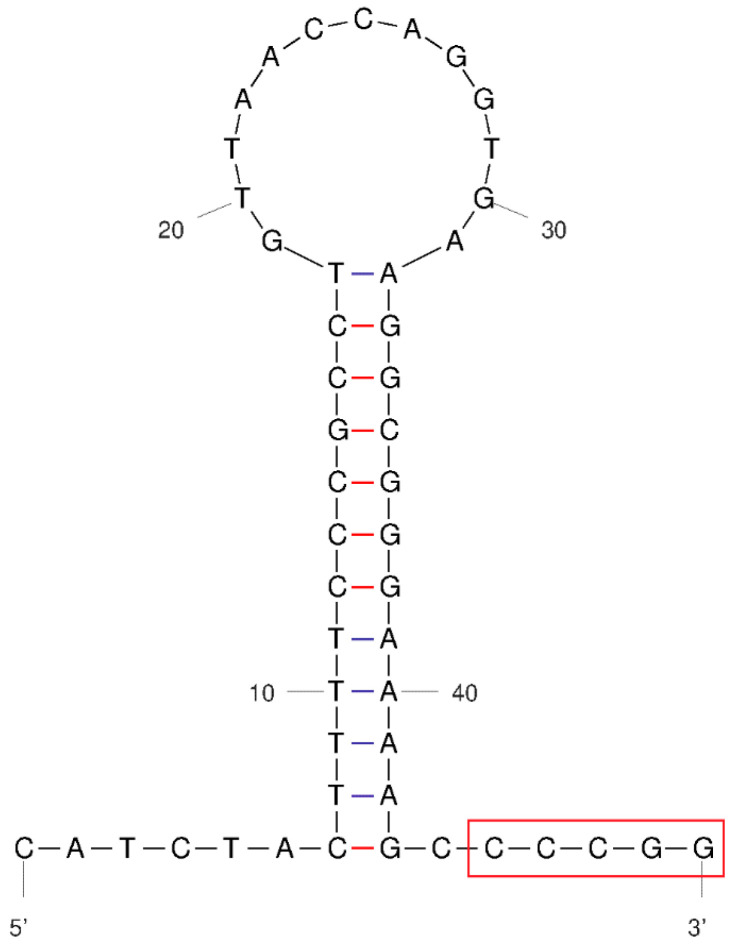
Stem-loop structure of O_L_ region in the mitogenome of *L. merzbacheri.* The conserved sequence region at the end of the stem is boxed.

The non-coding regions of mtDNA in *L. merzbacheri* included the O_L_ region and D-loop region. The O_L_ region resided between tRNA-Asn and tRNA-Cys with a whole length of 37 bp. The secondary structure of the O_L_ region was inferred using the Mfold tool (Figure 3). It consisted of 13 nt in the loop and 12 bp in the stem. The numbers of C and G in the stem were higher than those of A and T, while the reverse applied in the loop region, showing an obvious base asymmetry in this stem–loop structure. Similar to other vertebrates, the conserved sequence motif 3′-GGCCC-5′ was present adjacent to the 3′ end of the stem.

The length of entire mtDNA control region of *L. merzbacheri* was 929 bp. In this article, we recognized one extended termination associated sequence (ETAS), three enteral conserved elements (CSB-F, CSB-E and CSB-D), and three conserved elements (CSB-1, CSB-2 and CSB-3), respectively (Supplementary Figure S4). The ETAS was related to the synthesis termination of the D-loop, with a total length of 237 bp and the highest nucleotide variance rate. A termination-associated sequence ‘TACAT’ (53–57 bp) and its reverse complementary sequence ‘ATGTA’ (119–123 bp) were the key sequences of the ETAS domain, which made it form a stable hairpin structure. Meanwhile, the CD domain (333 bp) was the most conserved domain, and its typical central conserved block CSB-F was considered the distinguishing mark between ETAS and central CSB. CSB-1, CSB-2 and CSB-3 were found at the 3′-end of the control region with their iconic sequences 5′-CATCATTGAAAGACATA-3′, 5′-CAAACCCCCCTACCCCC-3′ and 5′-TGTCAAACCCCGAAACCAA-3′, respectively. CSB-1 was at the start of the conserved sequence block and was relatively less conserved than CSB-2 and CSB-3.

### 3.5. Phylogenetic Relationships of L. merzbacheri

**Figure 4:**
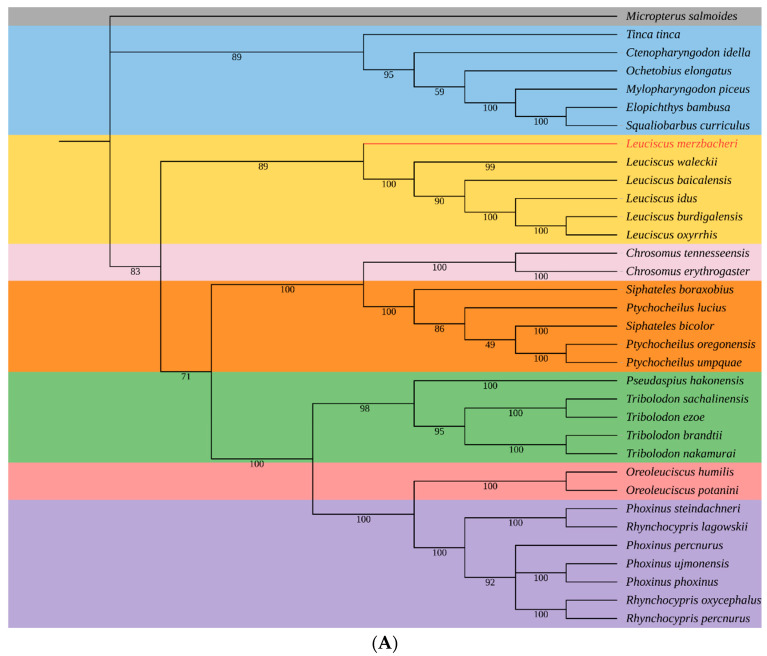

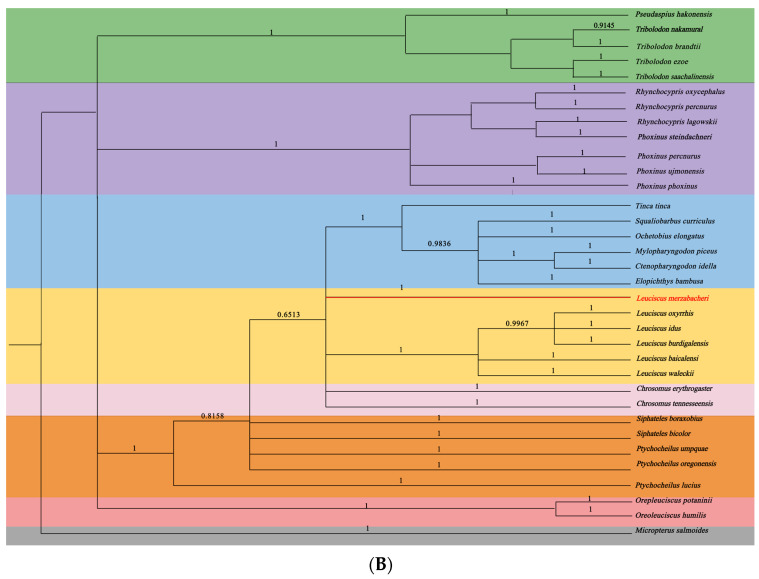
The ML (**A**) and BI (**B**) phylogenetic trees based on 13 concatenated PCG sequences. Numbers in the branches correspond to the bootstrap values (ML tree) and posterior probabilities (BI tree), respectively.

Two phylogenetic trees were constructed to analyze the evolutionary position of *L. merzbacheri* (Figure 4). The ML tree and BI tree exhibited similar topological structures, with species under the same taxonomic category roughly clustering into one clade. In the ML tree, *L. oxyrrhis* and *L. burdigalensis* first gathered together, and other *Leuciscus* species sequentially clustered outside. The BI tree was slightly different, with *L. oxyrrhis*, *L. idus* and *L. burdigalensis* clustering as a clade initially. A high bootstrap value and posterior probability were found in these two trees in which *L. merzbacheri* was located at the outermost level of the genus *Leuciscus* clade.

## 4. Discussion

The double-stranded circular mtDNA structure of *L. merzbacheri* was congruent with those of other vertebrates, and the total length was within the published length range of the Leuciscinae fishes’ mitochondrial genomes (15,672–17,865 bp). A positive AT-skew value and a negative GC-skew value were detected, reflecting the AT richness of the mitochondrial genome as a whole [43]. Among 13 PCGs, seven of them ended with incomplete stop codons TA- or T--. The truncated stop codons were usual in the mitogenomes of teleosts [44] and might be modified to the TAA during the mitochondrial mRNA 3′ polycistronic transcription and subsequent post-transcriptional polyadenylation [45].

The RSCU value means the number of occurrences of that codon in the genome. A value larger than 1.0 indicates that a given synonymous codon is preferred over the rest, less than 1.0 suggests a disfavored codon, and equivalent to 1.0 denotes no preference [46]. A total of 30 codons with RSCU values less than 1 were detected in all PCGs. Among them, the RSCU values of NNA codons were all greater than 1.0 only excluding UUA, which was positively correlated with the highest percentage of A at the third codon positions.

There were two copies of tRNA-Leu and tRNA-Ser in the mitogenome of *L. merzbacheri*, with the anti-codons UAA and UAG in the former, as well as the anti-codons UGA and GCU in the latter, respectively. It was speculated that the process of duplication, anticodon mutation and deletion of tRNA-Leu genes occurred during the evolution of the metazoan, and these two genes might have evolved independently for some time [47]. Only tRNA-Ser ^AGN^ could not form the cloverleaf structure, just like many other bony fishes [48]. The phenomenon of a wobble base pair was quite common in tRNAs [49]. It was indicated that the base pair of swinging pairs accounts for approximately 7% of the tRNA stem regions in fish mitochondria [44]. Herein, the noncanonical base pair G-U had the highest frequency. It was deduced that G-U mispairing might be biologically relevant with nucleic acid secondary structures and the amino acid misincorporation [50,51] and could be repaired and corrected through RNA editing [52].

As indispensable components of ribosomes, rRNAs play an important role in life activities, and many studies have shown that the functions of RNA are closely related to their secondary structures [53]. In this study, the two-dimensional structure of 16S rRNA was much more complex than that of 12S rRNA, which suggested that the latter was more conserved than the former. Therefore, 12S rRNA has been regarded as a DNA meta-barcode for the interspecific identification of fishes [54]. Moreover, a conserved multibranched loop (CML) was found in domain VI of 16S rRNA, which was the core of the ribosomal peptidyl transferase center (PTC) in prokaryotic 23S rRNA, a homologue to mtDNA 16S rRNA [55]. It was speculated that it is functionally important in catalyzing chemical reactions of protein synthesis [44].

A recent study indicated that an East Asian group of the subfamily Leuciscinae evolved independently, distant from those in Europe, Siberia and North America [28]. *Leuciscus* is a monophyletic group [56], and the divergence time of *L. merzbacheri* was estimated to be earlier than other daces in China [19,57]. It was confirmed that *L. idus* and *L. baicalensis* were more closely related to each other, and *L. merzbacheri* was the most ancient species among three *Leuciscus* fishes in Xinjiang [16,17]. Geographic isolation plays an important role in biological evolution and speciation [58]. The Junggar Basin is the second largest inland basin in China, which is surrounded by the Tianshan Mountains, the Altai Mountains and the Junggar Boundary Mountains, and the Gurbantungu Desert is located in its center. In addition, this region is dominated by a typical continental arid climate with an average annual temperature of 3–7 °C and yearly precipitation of 200 mm, respectively [59]. The natural geographical barrier, coupled with drought and a rainless environment allow a species to evolve in its own unique direction, which possibly led to a notable heredity difference between *L. merzbacheri* and congeneric species.

## 5. Conclusions

In the current study, a total of a 16,609 bp mitogenome sequence of *L. merzbacheri* was obtained based on a high-throughput sequencing technique, with the gene positions and features consistent with those of the published mitochondrial genomes of *Leuciscus* fishes. The overall nucleotide composition showed a clear AT preference, which is very common in the mitogenomes of most bony fishes. The phylogenetic analysis revealed that *Leuciscus* has a monophyletic origin. *L. merzbacheri* was well clustered with other *Leuciscus* fishes, and it was perhaps the earliest diverging species of this clade. The complete mitogenome of *L. merzbacheri* would be a useful resource for studies on the molecular taxonomy, species identification and genetic diversity of the genus *Leuciscus*.

## Figures and Tables

**Table 1:** Structural characteristics of the mitochondrial genome of *L. merzbacheri*.

Genes	Strand	Initiation Site	Termination Site	Length	Spacer (+)/Overlap (−)	Initiation Codon	Termination Codon	Anti-Codon
tRNA-Phe	H	1	69	69	0			GAA
12S rRNA	H	70	1028	959	0			
tRNA-Val	H	1029	1100	72	0			UAC
16S rRNA	H	1101	2791	1691	0			
tRNA-Leu ^UUR^	H	2792	2867	76	0			UAA
*ND1*	H	2868	3842	975	−2	ATG	TAG	
tRNA-Ile	H	3847	3918	72	4			GAU
tRNA-Gln	L	3917	3987	71	−2			UUG
tRNA-Met	H	3989	4057	69	1			CAU
*ND2*	H	4058	5102	1045	0	ATG	T	
tRNA-Trp	H	5103	5173	71	0			UCA
tRNA-Ala	L	5175	5243	69	1			UGC
tRNA-Asn	L	5245	5317	73	1			GUU
O_L_	H	5318	5354	37	0			
tRNA-Cys	L	5352	5418	67	−3			GCA
tRNA-Tyr	L	5420	5490	71	1			GUA
*COI*	H	5492	7042	1551	1	GTG	TAA	
tRNA-Ser ^UCN^	L	7043	7113	71	0			UGA
tRNA-Asp	H	7117	7190	74	3			GUC
*COII*	H	7204	7894	691	13	ATG	T	
tRNA-Lys	H	7895	7970	76	0			UUU
*ATP8*	H	7972	8136	165	1	ATG	TAG	
*ATP6*	H	8130	8812	683	−7	ATG	TA	
*COIII*	H	8813	9596	784	0	ATG	T	
tRNA-Gly	H	9597	9668	72	0			UCC
*ND3*	H	9669	10,017	349	0	ATG	T	
tRNA-Arg	H	10,018	10,087	70	0			UCG
*ND4L*	H	10,088	10,384	297	0	ATG	TAA	
*ND4*	H	10,378	11,759	1382	−7	ATG	TA	
tRNA-His	H	11,760	11,828	69	0			GUG
tRNA-Ser ^AGY^	H	11,829	11,897	69	0			GCU
tRNA-Leu ^CUN^	H	11,899	11,971	73	1			UAG
*ND5*	H	11,972	13,807	1836	0	ATG	TAG	
*ND6*	L	13,804	14,325	522	−4	ATG	TAA	
tRNA-Glu	L	14,326	14,394	69	0			UUC
Cyt *b*	H	14,399	15,539	1141	4	ATG	T	
tRNA-Thr	H	15,540	15,611	72	0			UGU
tRNA-Pro	L	15,611	15,680	70	−1			UGG
D-loop	H	15,681	16,609	929	0			

H: heavy-strand; L: light-strand; the negative number indicates the overlap, and superscript means the codon family.

**Table 2:** Frequency of codon usage in 13 PCGs of the *L. merzbacheri* mitogenome.

Codon	Count	RSCU	Codon	Count	RSCU	Codon	Count	RSCU	Codon	Count	RSCU
UUU(F)	95	0.84	**UCU(S)**	**48**	**1.20**	UAU(Y)	55	0.97	**UGU(C)**	**14**	**1.08**
**UUC(F)**	**132**	**1.16**	**UCC(S)**	**59**	**1.47**	**UAC(Y)**	**58**	**1.03**	UGC(C)	12	0.92
UUA(L)	103	0.99	**UCA(S)**	**64**	**1.59**	UAA(*)	0	0.00	**UGA(W)**	**85**	**1.43**
UUG(L)	45	0.43	UCG(S)	13	0.32	UAG(*)	0	0.00	UGG(W)	34	0.57
**CUU(L)**	**118**	**1.13**	CCU(P)	33	0.62	CAU(H)	27	0.52	CGU(R)	7	0.37
**CUC(L)**	**107**	**1.03**	**CCC(P)**	**77**	**1.44**	**CAC(H)**	**76**	**1.48**	CGC(R)	15	0.79
**CUA(L)**	**191**	**1.84**	**CCA(P)**	**82**	**1.53**	**CAA(Q)**	**68**	**1.39**	**CGA(R)**	**36**	**1.89**
CUG(L)	60	0.58	CCG(P)	22	0.41	CAG(Q)	30	0.61	CGG(R)	18	0.95
**AUU(I)**	**161**	**1.22**	ACU(T)	52	0.70	AAU(N)	45	0.79	AGU(S)	17	0.42
AUC(I)	102	0.78	**ACC(T)**	**106**	**1.42**	**AAC(N)**	**69**	**1.21**	**AGC(S)**	**40**	**1.00**
**AUA(M)**	**88**	**1.02**	**ACA(T)**	**105**	**1.41**	**AAA(K)**	**48**	**1.23**	AGA(*)	0	0.00
AUG(M)	85	0.98	ACG(T)	35	0.47	AAG(K)	30	0.77	AGG(*)	0	0.00
GUU(V)	59	0.90	GCU(A)	56	0.66	GAU(D)	32	0.82	GGU(G)	34	0.55
GUC(V)	63	0.96	**GCC(A)**	**149**	**1.75**	**GAC(D)**	**46**	**1.18**	**GGC(G)**	**66**	**1.06**
**GUA(V)**	**98**	**1.49**	**GCA(A)**	**103**	**1.21**	**GAA(E)**	**63**	**1.24**	**GGA(G)**	**84**	**1.35**
GUG(V)	43	0.65	GCG(A)	32	0.38	GAG(E)	39	0.76	**GGG(G)**	**64**	**1.03**

Preference codons are indicated in bold. Asterisk means the stop codon in mitochondrial DNA.

## Data Availability

The data presented in this study are openly available in the GenBank of NCBI (https://www.ncbi.nlm.nih.gov; accessed on 7 January 2024) under the accession number OR992052.

## Supplementary Materials

The following supporting information can be downloaded at: https://www.mdpi.com/article/10.3390/genes15101284/s1, Table S1: Information on the complete mitogenome sequences used in this study; Table S2: The nucleotide compositions and skewness of the *L. merzbacheri* mitogenome; Figure S1: Map of the mitochondrial genome of *L. merzbacheri*; Figure S2: The amino acid composition in 13 PCGs of the *L. merzbacheri* mitogenome; Figure S3: Secondary structures of rRNAs in the mitochondrial DNA of *L. merzbacheri*. (**a**) 12S rRNA. (**b**) 16S rRNA. Black bases indicate that they are the same as the template DNA, green bases indicate they are different from the template DNA, red bases indicate the inserted bases and blue bases indicate the reinsertion bases; Figure S4: Structural characteristics of the control region in *L. merzbacheri*.
