# Supplementary material for: Unraveling the Mitogenomic Characteristics and Phylogenetic Implications of Leuciscus merzbacheri (Zugmayer, 1912), an Endangered Fish in the Junggar Basin of Xinjiang, Northwest China
Source: Genes (Basel). 2024 Sep 30;15(10):1284. doi: 10.3390/genes15101284 (PMC11507045; doi:10.3390/genes15101284)
Supplement: Supplementary file 1 [file genes-15-01284-s001.zip › genes-3230451-supplementary.pdf]

Supplementary Materials

Table S1. Information of the complete mitogenome sequences used in this study.

Genus	Species	GenBank accession No.	Length (bp)
Chrosomus	*Chrosomus erythrogaster*	OR002152	16,599
	Chrosomus tennesseensis	MZ097372	16,596
Cetenopharyngodon	*Ctenopharyngodon idella*	MG827396	16,609
Elopichthys	*Elopichthys bambusa*	KM196112	16,619
Leuciscus	*Leuciscus baicalensis*	KF673863	16,606
	Leuciscus burdigalensis	KT223568	16,607
	Leuciscus idus	MT584106	16,603
	Leuciscus merzbacheri	This study	16,609
	Leuciscus oxyrrhis	KT223567	16,607
	Leuciscus waleckii	JX573111	16,605
Mylopharyngodon	*Mylopharyngodon piceus*	MF687109	16,614
Ochetobius	*Ochetobius elongatus*	KM400625	16,613
Oreoleuciscus	*Oreoleuciscus potanini*	AB626851	16,602
	Oreoleuciscus humilis	KJ754935	16,606
Phoxinus	*Phoxinus percnurus*	AP009149	16,600
	Phoxinus phoxinus	AB671170	17,865
	Phoxinus steindachneri	AP009148	16,597
	Phoxinus ujmonensis	KJ000673	17,738
Pseudaspius	*Pseudaspius hakonensis*	AB626855	16,602
Ptychocheilus	*Ptychocheilus oregonensis*	OK376045	16,605
	Ptychocheilus umpquae	OK623664	16,605
	Ptychocheilus lucius	MT501361	16,588
Rhynchocypris	*Rhynchocypris oxycephalus*	MK208924	16,583
	Rhynchocypris percnurus	KT359599	16,608
	Rhynchocypris lagowskii	AP009147	16,599
Siphateles	*Siphateles bicolor*	OL457397	16,601
	Siphateles boraxobius	MN296123	16,599
Squaliobarbus	*Squaliobarbus curriculus*	KC351187	16,619
Tinca	*Tinca tinca*	AB218686	16,612
Tibolodon	*Tribolodon ezoe*	AP011270	15,672
	Tribolodon sachalinensis	AB626856	16,597
	Tribolodon nakamurai	AB218896	16,616
	Tribolodon brandtii	AB626853	16,611

Table S2. The nucleotide compositions and skewness of *L. merzbacheri* mitogenome.

**Genes**	**T (%)**	**C (%)**	**A (%)**	**G (%)**	**A+T (%)**	**C+G (%)**	**AT-skew**	**GC-skew**
Mitogenome	26.30	27.12	27.90	18.68	54.20	45.80	0.0295	-0.1843
The 1st codon	21.51	25.46	25.88	27.15	47.39	52.61	0.0922	0.0321
The 2nd codon	40.81	27.28	18.06	13.85	58.87	41.13	-0.3864	-0.3265
The 3rd codon	22.46	30.99	32.07	14.48	54.53	45.47	0.1762	-0.3631
13 PCGs	28.26	27.91	25.34	18.49	53.60	46.40	-0.0545	-0.2030
ND1	28.00	29.95	22.26	19.79	50.26	49.74	-0.1142	-0.2043
ND2	25.65	30.81	24.31	19.23	49.96	50.04	-0.0268	-0.2314
ND3	30.09	26.93	25.50	17.48	55.59	44.41	-0.0826	-0.2128
ND4	28.36	27.72	26.05	17.87	54.41	45.59	-0.0425	-0.2161
ND4L	28.28	29.63	24.92	17.17	53.20	46.80	-0.0632	-0.2662
ND5	26.96	28.59	27.29	17.16	54.25	45.75	0.0061	-0.2498
ND6	14.37	33.52	35.44	16.67	49.81	50.19	0.4230	-0.3357
COI	28.82	27.01	25.15	19.02	53.97	46.03	-0.0680	-0.1736
COII	28.07	25.76	28.22	17.95	56.29	43.71	0.0027	-0.1787
COIII	29.85	26.65	26.15	17.35	56.00	44.00	-0.0661	-0.2114
ATP6	28.99	28.40	27.23	15.38	56.22	43.78	-0.0313	-0.2974
ATP8	24.85	27.88	36.36	10.91	61.21	38.79	0.1880	-0.4375
Cytb	27.78	30.07	25.59	16.56	53.37	46.63	-0.0410	-0.2897
12S rRNA	19.81	25.86	31.18	23.15	50.99	49.01	0.2230	-0.0553
16S rRNA	21.17	22.95	33.47	22.41	54.64	45.36	0.2251	-0.0119
D-loop	31.54	21.96	31.22	15.28	62.76	37.24	-0.0051	-0.1794

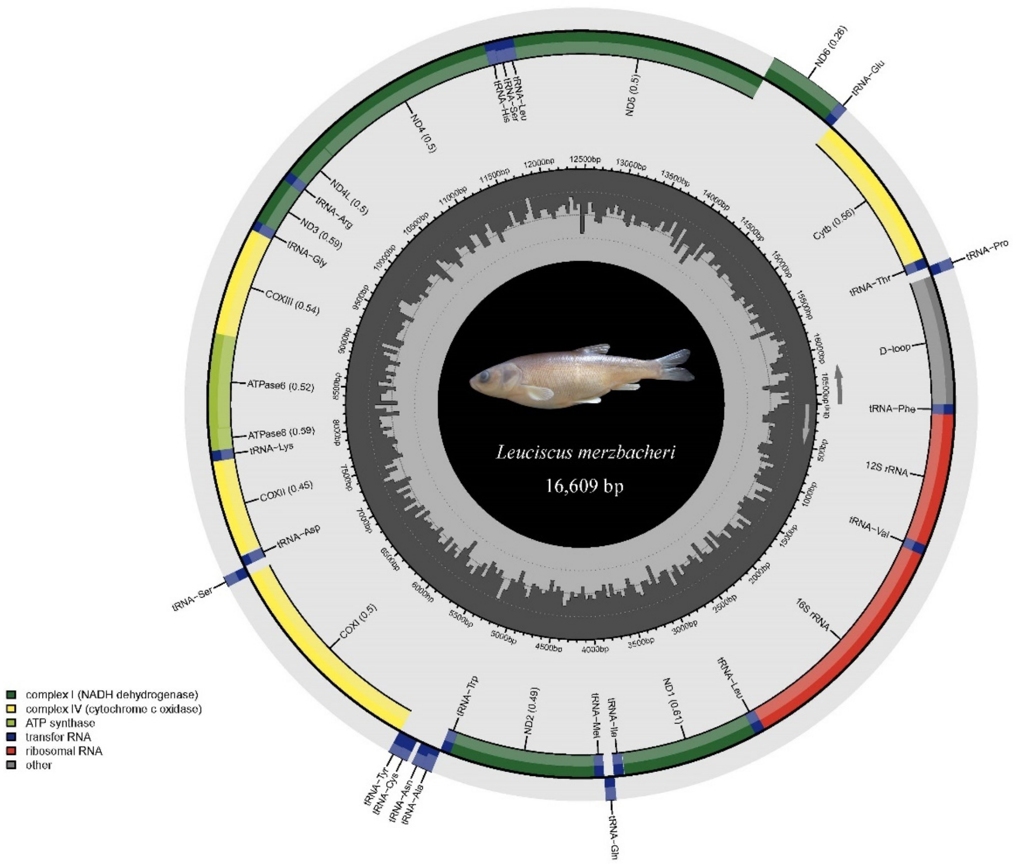

Figure S1. Map of the mitochondrial genome of *L. merzbacheri.*

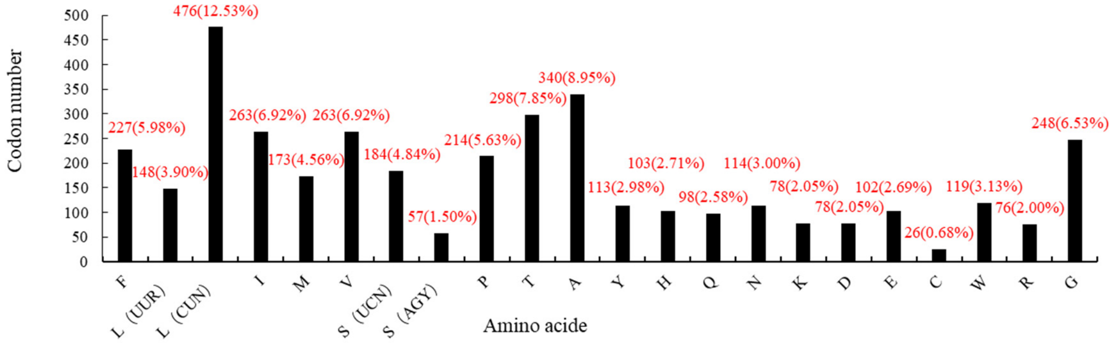

Figure S2. The amino acid composition in 13 PCGs of *L. merzbacheri* mitogenome.

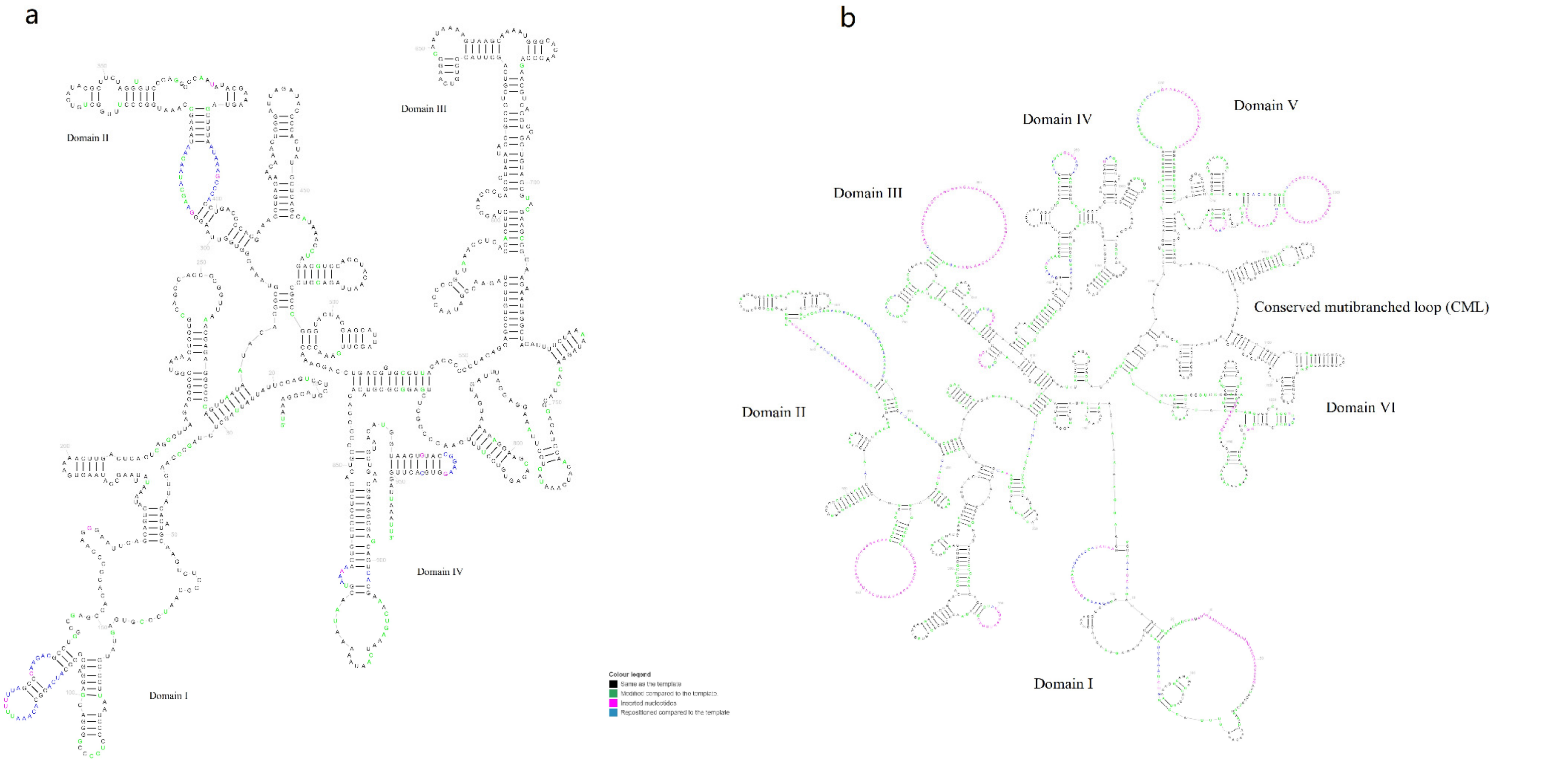

Figure S3. Secondary structures of rRNAs in the mitochondrial DNA of *L. merzbacheri*. **(a)** 12S rRNA. **(b)**16S rRNA. Black bases indicate they are the same as the template DNA, green bases indicate they are different from the template DNA, red bases indicate the inserted bases and blue bases indicate the reinsertion bases.

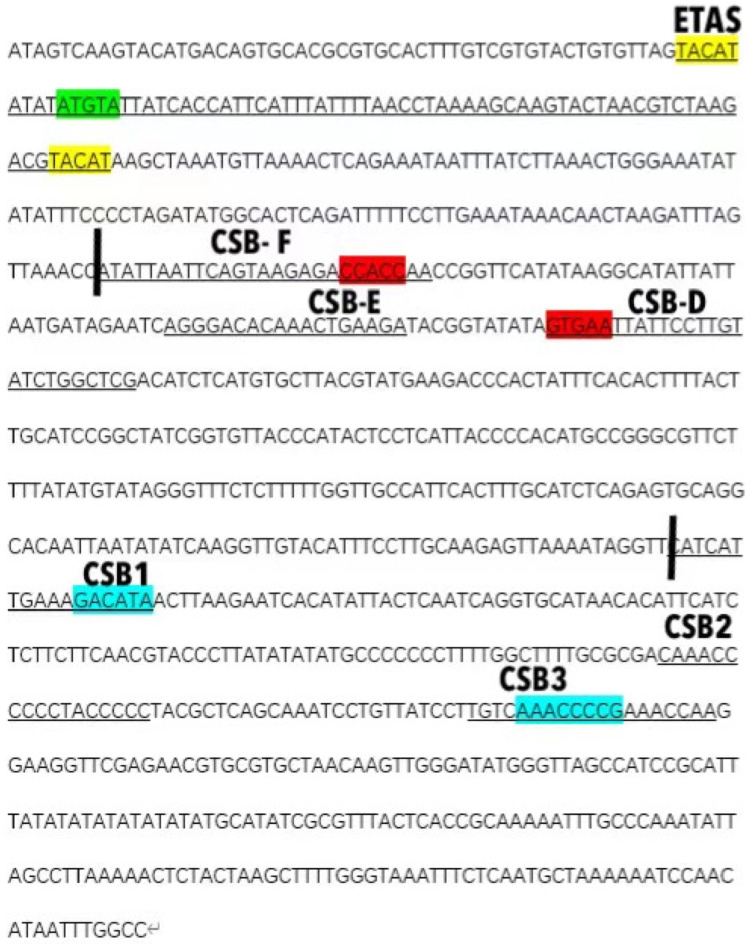

Figure S4. Structural characteristics of the control region in *L. merzbacheri.*
